# Supplementary material for: The effect of photoperiod, environmental temperature and wind speed on external quality of free-range turkey eggs
Source: PLoS One. 2025 Jun 18;20(6):e0326308. doi: 10.1371/journal.pone.0326308 (PMC12176130; doi:10.1371/journal.pone.0326308)
Supplement: S1 Table — (DOCX) [file pone.0326308.s001.docx]

S1 Table. **Mean values of weather attributes and moon phase for each egg category.**

| Class \ Variable | Sharp-1 | Sharp-2 | Sharp-3 | Standard-1 | Standard-2 | Standard-3 | Rounded-3 |
| --- | --- | --- | --- | --- | --- | --- | --- |
| Sunshine  hours (h) | 11.19 | 8.14 | 6.21 | 12.02 | 8.91 | 6.00 | 5.33 |
| Minimum temperature (°C) | 13.64 | 9.06 | 8.99 | 13.05 | 9.31 | 9.78 | 8.23 |
| Maximum gust speed (m/s) | 11.26 | 10.36 | 9.35 | 10.37 | 10.13 | 9.14 | 8.12 |
| Rainfall (l/m^2^) | 2.64 | 5.23 | 4.29 | 0.39 | 3.21 | 2.93 | 0.58 |
| Moon illuminated (%) | 40.26 | 57.84 | 58.39 | 54.48 | 58.87 | 55.82 | 62.17 |
| Average wind speed (m/s) | 4.12 | 3.04 | 2.84 | 4.38 | 2.97 | 3.23 | 2.69 |
| Wind  direction (°) | 39.74 | 51.12 | 49.81 | 48.23 | 59.33 | 46.76 | 52.42 |
| Maximum pressure (mB) | 1006.91 | 1007.51 | 1007.06 | 1007.55 | 1007.23 | 1007.29 | 1008.35 |
