# Supplementary material for: The effect of photoperiod, environmental temperature and wind speed on external quality of free-range turkey eggs
Source: PLoS One. 2025 Jun 18;20(6):e0326308. doi: 10.1371/journal.pone.0326308 (PMC12176130; doi:10.1371/journal.pone.0326308)
Supplement: Attachment — Submitted filename: PONE-D-25-17693.pdf [file pone.0326308.s002.pdf]

PLOS ONE

The effect of photoperiod, environmental temperature and wind speed on external quality of free-range turkey eggs

--Manuscript Draft--

**Manuscript Number:**	PONE-D-25-17693
**Article Type:**	Research Article
**Full Title:**	The effect of photoperiod, environmental temperature and wind speed on external quality of free-range turkey eggs
**Short Title:**	Influence of weather on turkey egg quality
**Corresponding Author:**	Antonio González Ariza<br>Diputación de Córdoba<br>Córdoba, SPAIN
**Keywords:**	Alternative production systems, cold stress, egg weight, photoperiod, shape index.
**Abstract:**	The present study aimed to determine the influence of meteorological events and the moon cycle on the weight and shape index of turkey eggs produced in alternative systems. To this aim, 194 eggs laid by Andalusian turkey hens raised outdoors were collected and daily measured for 14 months. Seven categories were obtained attending to their weight (heavy, medium, and light) and shape index (sharp, standard, and round), and performed as dependent variables in the discriminant canonical analysis (DCA). As explanatory variables, meteorological and moon cycle features were collected from repositories consulting the day before each egg was laid. Minimum pressure, moon phase, and maximum temperature reported multicollinearity problems (VIF > 5.0) and were removed from further analysis. Moderate correlations among climatic variables influencing egg quality were found and ranged from + 0.574 to – 0.537, involving maximum gust speed in both cases. Variables exhibiting explanatory power in the DCA were sunshine hours (λ = 0.691, F = 13.950), minimum temperature (λ = 0.874, F = 4.476), and maximum gust speed (λ = 0.944, F = 1.841). Results evidence alternative productions present a different environmental exposure than indoors, as well as highlight the suitability of this breed for free range systems.
**Order of Authors:**	José Ignacio Salgadop Pardo
	Juan Vicente Delgado Bermejo
	Antonio González Ariza
	José Manuel León Jurado
	María Esperanza Camacho Vallejo
**Additional Information:**	
**Question**	**Response**
**Financial Disclosure**<br><br>Enter a financial disclosure statement that describes the sources of funding for the work included in this submission. Review the submission guidelines for detailed requirements. View published research articles from *PLOS ONE* for specific examples.<br><br>This statement is required for submission and **will appear in the published article** if the submission is accepted. Please make	Yes

Powered by Editorial Manager® and ProduXion Manager® from Aries Systems Corporation

sure it is accurate.<br><br>**Funded studies**<br>Enter a statement with the following details:<br>• Initials of the authors who received each award<br>• Grant numbers awarded to each author<br>• The full name of each funder<br>• URL of each funder website<br>• Did the sponsors or funders play any role in the study design, data collection and analysis, decision to publish, or preparation of the manuscript?<br><br>Did you receive funding for this work?	
Please add funding details.<br>as follow-up to "**Financial Disclosure**<br><br>Enter a financial disclosure statement that describes the sources of funding for the work included in this submission. Review the submission guidelines for detailed requirements. View published research articles from *PLOS ONE* for specific examples.<br><br>This statement is required for submission and **will appear in the published article** if the submission is accepted. Please make sure it is accurate.<br><br>**Funded studies**<br>Enter a statement with the following details:<br>• Initials of the authors who received each award<br>• Grant numbers awarded to each author<br>• The full name of each funder<br>• URL of each funder website<br>• Did the sponsors or funders play any role in the study design, data collection and analysis, decision to publish, or preparation of the manuscript?<br><br>Did you receive funding for this work?"	This research was funded by FEDER project P20_00893 and, during the covering period of a pre-doctoral contract (FPU Fellowship, with code FPU21/00010), the Spanish Ministry of Science and Innovation.
Please select the country of your main research funder (please select carefully	SPAIN - ES

as in some cases this is used in fee calculation).<br>as follow-up to "**Financial Disclosure**<br><br>Enter a financial disclosure statement that describes the sources of funding for the work included in this submission. Review the submission guidelines for detailed requirements. View published research articles from *PLOS ONE* for specific examples.<br><br>This statement is required for submission and **will appear in the published article** if the submission is accepted. Please make sure it is accurate.<br><br>**Funded studies**<br>Enter a statement with the following details:<br>• Initials of the authors who received each award<br>• Grant numbers awarded to each author<br>• The full name of each funder<br>• URL of each funder website<br>• Did the sponsors or funders play any role in the study design, data collection and analysis, decision to publish, or preparation of the manuscript?<br><br>Did you receive funding for this work?"	
**Competing Interests**<br><br>Use the instructions below to enter a competing interest statement for this submission. On behalf of all authors, disclose any competing interests that could be perceived to bias this work—acknowledging all financial support and any other relevant financial or non-financial competing interests.<br><br>This statement is required for submission and **will appear in the published article** if the submission is accepted. Please make sure it is accurate and that any funding sources listed in your Funding Information later in the submission form are also	The authors have declared that no competing interests exist.

declared in your Financial Disclosure statement.<br><br>View published research articles from *PLOS ONE* for specific examples.<br><br>**NO authors have competing interests**<br><br>Enter: *The authors have declared that no competing interests exist.*<br><br>**Authors with competing interests**<br><br>Enter competing interest details beginning with this statement:<br><br>*I have read the journal's policy and the authors of this manuscript have the following competing interests: [insert competing interests here]*<br><br>* typeset	
**Ethics Statement**<br><br>Enter an ethics statement for this submission. This statement is required if the study involved:<br><br>• Human participants<br>• Human specimens or tissue<br>• Vertebrate animals or cephalopods<br>• Vertebrate embryos or tissues<br>• Field research<br><br>Write "N/A" if the submission does not require an ethics statement.<br><br>General guidance is provided below. Consult the submission guidelines for detailed instructions. **Make sure that all information entered here is included in the Methods section of the manuscript.**	The present work was performed according to the Declaration of Helsinki, the Directive 2010/63/EU of the European Parliament and of the Council of September 22, and the Spanish legislation (Royal Decree-national law 113/2013, of February 1). The experimental phase of the study eluded the scope of evaluation of the Ethics Review Committee of the University of Córdoba, as it did not comply with the legislation on animal protection in experimentation. The methods developed were under the ARRIVE guidelines and the authority of the Agropecuary Provincial Centre of Diputación de Córdoba (the Córdoba Provincial Council; Spain), where the study was conducted, granted permission.

**Format for specific study types**<br><br>**Human Subject Research (involving human participants and/or tissue)**<br>• Give the name of the institutional review board or ethics committee that approved the study<br>• Include the approval number and/or a statement indicating approval of this research<br>• Indicate the form of consent obtained (written/oral) or the reason that consent was not obtained (e.g. the data were analyzed anonymously)<br><br>**Animal Research (involving vertebrate animals, embryos or tissues)**<br>• Provide the name of the Institutional Animal Care and Use Committee (IACUC) or other relevant ethics board that reviewed the study protocol, and indicate whether they approved this research or granted a formal waiver of ethical approval<br>• Include an approval number if one was obtained<br>• If the study involved *non-human primates*, add *additional details* about animal welfare and steps taken to ameliorate suffering<br>• If anesthesia, euthanasia, or any kind of animal sacrifice is part of the study, include briefly which substances and/or methods were applied<br><br>**Field Research**<br><br>Include the following details if this study involves the collection of plant, animal, or other materials from a natural setting:<br>• Field permit number<br>• Name of the institution or relevant body that granted permission	
**Data Availability**<br><br>Authors are required to make all data underlying the findings described fully available, without restriction, and from the time of publication. PLOS allows rare exceptions to address legal and ethical concerns. See the PLOS Data Policy and FAQ for detailed information.	Yes - all data are fully available without restriction

A Data Availability Statement describing where the data can be found is required at submission. Your answers to this question constitute the Data Availability Statement and **will be published in the article**, if accepted.<br><br>**Important:** Stating 'data available on request from the author' is not sufficient. If your data are only available upon request, select 'No' for the first question and explain your exceptional situation in the text box.<br><br>Do the authors confirm that all data underlying the findings described in their manuscript are fully available without restriction?	
**Describe where the data may be found in full sentences. If you are copying our sample text, replace any instances of XXX with the appropriate details.**<br><br>• If the data are **held or will be held in a public repository**, include URLs, accession numbers or DOIs. If this information will only be available after acceptance, indicate this by ticking the box below. For example: *All XXX files are available from the XXX database (accession number(s) XXX, XXX.*).<br>• If the data are all contained **within the manuscript and/or Supporting Information files**, enter the following: *All relevant data are within the manuscript and its Supporting Information files.*<br>• If neither of these applies but you are able to provide **details of access elsewhere**, with or without limitations, please do so. For example:<br><br>*Data cannot be shared publicly because of [XXX]. Data are available from the XXX Institutional Data Access / Ethics Committee (contact via XXX) for researchers who meet the criteria for access to confidential data.*<br><br>*The data underlying the results presented in the study are available from (include the name of the third party*	All relevant data are within the manuscript and its Supporting Information files.

and contact information or URL).<br>• This text is appropriate if the data are owned by a third party and authors do not have permission to share the data.<br><br>* typeset	
Additional data availability information:	

Powered by Editorial Manager® and ProduXion Manager® from Aries Systems Corporation

Manuscript
Click here to access/download;Manuscript;Manuscript figures deleted 2025-04-04.docx

The effect of photoperiod, environmental temperature and wind speed on external quality of free-range turkey eggs

Influence of weather on turkey egg quality

José Ignacio Salgado Pardo [1], Juan Vicente Delgado Bermejo [1], Antonio González Ariza [2]*, José Manuel León Jurado [2], María Esperanza Camacho Vallejo [3]

[1] Department of Genetics, Faculty of Veterinary Sciences, University of Córdoba, Córdoba, Spain

[2] Agropecuary Provincial Center of Córdoba, Provincial Council of Córdoba, Córdoba, Spain

[3] Department of Agriculture and Ecological Husbandry, Area of Agriculture and Environment, Andalusian Institute of Agricultural and Fisheries Research and Training (IFAPA), Alameda del Obispo, Córdoba, Spain

* Corresponding author: Antonio González Ariza.

Email: angoarvet@outlook.es

Abstract

The present study aimed to determine the influence of meteorological events and the moon cycle on the weight and shape index of turkey eggs produced in alternative systems. To this aim, 194 eggs laid by Andalusian turkey hens raised outdoors were collected and daily measured for 14 months. Seven categories were obtained attending to their weight (heavy, medium, and light) and shape index (sharp, standard, and round), and performed as dependent variables in the discriminant canonical analysis (DCA). As explanatory variables, meteorological and moon cycle features were collected from repositories consulting the day before each egg was laid. Minimum pressure, moon phase, and maximum temperature reported multicollinearity problems (VIF > 5.0) and were removed from further analysis. Moderate correlations among climatic variables influencing egg quality were found and ranged from + 0.574 to – 0.537, involving maximum gust speed in both cases. Variables exhibiting explanatory power in the DCA were sunshine hours (λ = 0.691, F = 13.950), minimum temperature (λ = 0.874, F = 4.476), and maximum gust speed (λ = 0.944, F = 1.841). Results evidence alternative productions present a different environmental exposure than indoors, as well as highlight the suitability of this breed for free range systems.

Introduction

Alternative poultry production has arisen in developed countries during the last decades [1-3] due to the market's demands for natural products obtained from sustainable and animal welfare-friendly systems [4]. In this context, traditional farming as backyard and extensive regimes are being restored [5]. In addition to the food contribution, alternative farming has a positive impact on overall biodiversity [6], the preservation of local breeds, and the fixation of rural populations [1, 7]. This situation contrasts dramatically with that of developing countries, where backyard production is not a preference, but a necessity. Depending on the region, backyard poultry comprises about 30-80% of the total census [8] and can contribute up to 90% of poultry production [9, 10]. In addition, backyard poultry also plays an essential social and cultural role in these communities, providing a simple source of income [11-13].

In this context, turkey is one of the most widespread poultry species in the world [14] despite its relatively young domestication compared to other bird species [15]. Its high heat tolerance and grazing aptitude have made this species a great option for backyard systems, even in arid and tropical regions [11, 16, 17]. However, little attention has been paid to turkey eggs as food, possibly due to their low natural suitability for laying [18]. Nevertheless, backyard turkey eggs are an important source of animal protein in some regions of South Asia [19], Nigeria [11, 20], and Mexico [13, 16]. On the other hand, in developed countries, turkey eggs are mainly allocated to hatching [21], and marketing channels have not been developed. The consumption of turkey eggs in these regions is marginal and concentrated in rural areas, based on local breeds reared in backyards [22, 23]. However, thanks to the particularities of the species [24], organic turkey eggs from local breeds are considered niche products with high potential in haute cuisine [25].

Despite this promising future, outdoor systems face high environmental exposure [26, 27], which accounts for up to 30% of the overall variance in external egg traits [28]. This situation is even more concerning in a changing world, where global warming threatens food security [29]. Weather is one of the main environmental factors influencing turkey performance, particularly temperature, humidity, and photoperiod [30]. In this line, high-yielding hybrid strains struggle in uncontrolled environments, leading to losses in productivity and welfare issues [2, 4]. On the other hand, indigenous genotypes are locally adapted, and their high rusticity and instincts make them more suitable for outdoor systems [31]. Although their lower productivity makes their products costly [4], consumers who are conscious of the great quality of native breed products are willing to pay [2, 5, 32]. In this context, few adapted turkey landraces have been described in the Mediterranean basin, where extreme climatic conditions are reached [33, 34]. To this respect, the Andalusian turkey is a light and rustic population from the south of Spain commonly reared in backyards and pasture regimes [35]. Although this genotype could be direct descendants of the first turkeys to arrive in Europe in the 16th century [17, 23], they are currently in a severely endangered status. Additionally, no protection figures have been developed for turkey breeds in Spain, since no native turkey landrace is included in the Spanish Ministry of Agriculture’s breed catalogue (Royal Decree-national law 527/2023, of June 20). In this context, the promotion of the consumption of close by, organic eggs to encourage the use of the breed and improve profitability has been proposed [7, 33].

To this aim, the productive characterization of this population is mandatory. However, resource allocation in the research of local genotypes is generally poor [31, 36]. This situation is even more remarkable in the turkey species, in which there is a remarkable lack of studies addressing egg quality in unconventional rearing systems [13]. For this reason, simple but cheap research approaches avoiding the breaking of the shell should be addressed. In this line, external

quality attributes could act as an approach to overall egg quality. In this regard, egg weight and shape index are considered among the external parameters most closely associated with internal egg quality [13, 37, 38]. They are strongly correlated with albumen [38-42] and yolk quality [13, 37, 39, 41-43], as well as other external attributes such as shell quality and porosity [40]. Additionally, literature has reported their association with overall hatchability [37, 44, 45] and their use for determining poult’s sex [35]. The association of egg total weight and shape index with overall egg quality, alongside their high heritability [43] has led to these two traits being included in selection schemes and evaluation of elite birds [46]. Therefore, the study of egg weight and shape index must be proposed as a first approach to studying overall egg quality when scarce resources are available.

Hence, the present work aims to determine the influence of meteorological conditions and the lunar cycle on the external quality of eggs laid by outdoor Andalusian turkey hens. This might enable the determination of the main environmental effects influencing egg quality in turkeys, which will lead to a deeper understanding of the interaction of the environment on alternative productions. This study is the pioneer in this field and will lead to further studies, as overall quality is approached from the study of egg weight and shape index. Additionally, the present work contributes to the productive characterization of the Andalusian turkey, which would contribute to the governmental recognition of the genetic resource as a breed.

Material and methods

Study design

Ethics approval

The present work was performed according to the Declaration of Helsinki, the Directive 2010/63/EU of the European Parliament and of the Council of September 22, and the Spanish legislation (Royal Decree-national law 113/2013, of February 1). The experimental phase of the study eluded the scope of evaluation of the Ethics Review Committee of the University of Córdoba, as it did not comply with the legislation on animal protection in experimentation. The methods developed were under the ARRIVE guidelines and the authority of the Agropecuary Provincial Centre of Diputación de Córdoba (the Córdoba Provincial Council; Spain), where the study was conducted, granted permission.

Flock housing and environmental conditions

The experimental phase of the study was carried out in the facilities of the Agropecuary Provincial Centre of Diputación de Córdoba, in Andalusia (Spain) from February 2019 to April 2020. Eggs included in the study were laid by a flock of mature turkey hens from the conservation nuclei of the Andalusian turkey breed. Animals were not involved in further experiments but in the egg control production, and were kept in outdoor pens, simulating backyard conditions. Ad libitum water and commercial feed (15.20% crude protein, 4.10% calcium, 0.66% available phosphorus) were provided to hens during the complete experimental phase.

Work sample and quality measurements

The sample comprised 194 eggs collected during the 14-month experimental phase, to obtain eggs laid during a complete year. Measures were taken in the 24 hours after oviposition. Egg weight was measured using an electronic scale (Cobos, CSB-600C, Barcelona, Spain; precision: ±0.01g.) The major diameter or long axis, considered as the maximum distance

between the broad and narrow poles, and the minor diameter or short axis, considered as the line of maximum width, were measured using a Vernier scale (Electro DH M 60.205, Barcelona, Spain). Diameters were employed to determine the shape index (SI) of the eggs, computed through the following formula:

$$SI = \frac{Øm}{ØM} \times 100,$$

where ØM is the major diameter and Øm is the minor diameter.

Meteorological variables and moon phase

Records were completed by collecting information on weather conditions and moon phases during the 24 hours before egg laying [47, 48]. Information about the moon phase was obtained from the Astronomical Applications Department of the US Naval Observatory website (https://aa.usno.navy.mil/ accessed on 12 February 2024; Coordinates: 37°91′51″ N–04°70′91″ W). Moon phases were registered distinguishing seven categories: waxing gibbous, waning crescent, waxing crescent, waning gibbous, new moon, first quarter, and full moon. Moreover, the percentage of the moon-illuminated surface was recorded as well. Weather parameters were obtained from the official, historical data repository of the meteorological station located near the study site (https://datosclima.es/ accessed on 12 February 2024; Coordinates: 37°50′56″ N–04°50′48″ W). Parameters included were maximum temperature (°C), minimum temperature (°C), maximum pressure (mB), minimum pressure (mB), maximum gust speed (m/s), wind direction (°), average wind speed (m/s), photoperiod (h) and daily rainfall (l/m^2).

Statistical analysis of data

Discriminant canonical analysis

A discriminant canonical analysis (DCA) was performed to develop a statistical tool enabling the classification of eggs according to the influence of meteorological and moon phase events on external egg quality. To this aim, eggs were grouped according to their weight and shape index. Weight terciles were created from database observations and categories were identified as heavy eggs (100.06 – 85.77 g, tercile 1), medium eggs (85.69 – 77.33 g, tercile 2), and light eggs (77.31 – 61.25 g, tercile 3). For shape index (SI), eggs were classified attending to commercial standards developed in chicken as sharp ($SI < 72$), standard ($72 < SI < 76$), and rounded ($SI > 76$) [1, 7]. Therefore, seven categories were obtained by combining egg weight and shape index: Sharp–1, Sharp–2, Sharp–3, Standard–1, Standard–2, Standard–3, and Rounded–3. No observations ascribed for the Rounded-1 and Rounded-2 categories were found in the database.

The egg weight and shape index combination were considered as the dependent variables in the DCA, while the aforementioned meteorological and moon phase parameters were included as explanatory variables. The canonical relationships among traits were graphically represented to exhibit group differences in a territorial map. Regularized forward stepwise multinomial logistic regression algorithms were used to perform the variable selection. Prior probability was computed to regularize priors according to the group sizes. All the analyses were run using the discriminant analysis routine of the Analyzing Data package of XLSTAT software (Addinsoft Pearson Edition 2014, Addinsoft, Paris, France).

Preliminary multicollinearity testing

Before the computation of discriminant canonical analysis, the multicollinearity assumption must be proved to detect redundancies. A redundant variable is one that explains the same source of variation as another already present in the study without contributing further

additional variation. The presence of redundant variables in the analysis means the overinflation of variability in the analysis. Multicollinearity represents the linear intercorrelation between just two variables or between one linear variable and the linear combination of others [1]. In order to determine multicollinearity among variables, the variance inflation factor (VIF) is a parameter commonly employed in statistical analysis due to its ability to detect the increase in variance of a regression coefficient due to collinearity. The variance inflation factor (VIF) and tolerance have been employed in many studies in animal science research [1, 7, 31, 48], following the formula:

$$VIF = \frac{1}{1 - R^2},$$

where R^2 represents the determination coefficient of the regression equation. Values under 5 are recommended to avoid multicollinearity problems in variables [7].

Discriminant canonical analysis efficiency and model reliability

To define variables significantly contributing to the discriminant function, the Wilks' Lambda test was performed. As values tend to 0, the greatest contribution of a variable to the discriminant function. However, only significance (χ^2) values under 0.05 are considered to explain group adscription [1].

Due to the lack of equality in sample sizes, Pillai's trace criterion was selected for the assumption of equal covariance matrices in the discriminant function analysis [49]. Obtaining a significance equal to or under 0.05 is indicative of the statistical significance of the set of predictors [7].

Variable Dimensionality Reduction

A principal component analysis was employed before performing a DCA. This enabled the identification of those few most contributing variables in the effect of environment on egg weight and shape index.

Canonical coefficients, loading interpretation, and special representation

The percentage of allocation of each observation within its group was determined through the discriminant function analysis. Thus, according to the bibliography, only discriminant loading values ≥|0.40| were considered to define substantive discriminating variables [34]. Variables exhibiting greater absolute coefficients within a set were those reporting a higher discriminant ability. Nonsignificant variables were removed before entering the function by the use of the stepwise procedure technique [50]. Subsequently, squared Mahalanobis distances and principal component analysis were performed using this formula:

$$D_{ij}^2 = \left(\bar{Y}_i - \bar{Y}_j\right) COV^{-1}\left(\bar{Y}_i - \bar{Y}_j\right),$$

where D_{ij}^2 represents the distance between population i and j; COV^{-1} is the inverse of the covariance matrix of measured variable x, and both $\bar{Y}_i$ and $\bar{Y}_j$ represent the means of variable x in the ith and jth populations, respectively.

The Mahalanobis distance matrix obtained was then transformed into an Euclidean distance matrix, and a dendrogram representing the clusters within the environmental classification of egg shape and size was plotted. This was performed using the underweighted pair-group method arithmetic averages (UPGMA) from the Universität Rovira i Virgili (URV), Tarragona, Spain, and the Phylogeny procedure of MEGA X 10.0.5 (Institute of Molecular Evolutionary Genetics, The Pennsylvania State University, State College, PA, USA).

Discriminant function cross-validation

To test the reliability of the discriminant function, the leave-one-out cross-validation was performed. This cross-validation recorded the proportion of the right assignment of eggs within their clusters (weight and shape index categories) based on the environmental factors registered for each observation. The relative distance of each observation to the centroid of its closest cluster was employed. Press` Q statistic was performed to compare the discriminating power of the cross-validated function, following the formula:

$$Press'Q = \frac{(N - nK)^2}{N(K-1)},$$

where N is the number of observations in the sample, n represents the number of correctly classified observations and K is the number of groups. If Press' Q statistic value exceeds χ^2 = 6.63, the cross-classification ability of the function is considered significantly better than chance.

Results

Preliminary multicollinearity test

Minimum pressure, maximum temperature and moon phase were found to be redundant variables (VIF > 5.0) and were discarded from further analysis. Parameters not showing multicollinearity problems are shown in Table 1.

Table 1. Multicollinearity preliminary test of environmental factors influencing egg weight and shape index.

Statistics/Parameters	Tolerance (1 - R^2)	VIF[a]
Maximum gust speed	0.279	3.584

Rainfall	0.310	3.226
Sunshine hours	0.374	2.677
Minimum temperature	0.490	2.040
Maximum pressure	0.528	1.892
% Moon illuminated	0.722	1.385
Wind direction	0.820	1.220
Average wind speed	0.823	1.215

[a] Variance inflation factor (VIF) = 1 (not correlated); 1 < VIF < 5 (moderately correlated); VIF ≥ 5 (highly correlated).

DCA model reliability

As shown in Table 2, the significance value of Pillai’s trace criterion evidenced significant differences across egg categories, and thus, justified the implementation of the DCA.

Table 2. Results of the Pillai’s trace test of equality of covariance matrices of the canonical discriminant functions allowing the implementation of the DCA.

Parameter	Value
Pillai’s Trace Criterion	0.523
F (Observed value)	2.208
F (Critical value)	1.369
df1	48
df2	1110
Significance	<0.0001
alpha	0.05

F = Snedecor’s F; df1 = numerator degrees of freedom for the F-approximation; df2 = denominator degrees of freedom for the F-approximation.

Canonical coefficients, loading interpretation, and spatial representation

Six canonical discriminating functions compose the DCA (Fig 1). The sum of the contributions of the first three functions accounts for 97.66% of the overall variance.

Fig 1. Canonical variable functions and percentages of cumulative variance.

Fig 2 represents the loading value of each dependent variable (environmental factors) in the three first discriminant functions.

Fig 2. Loading values of each environmental factor influencing egg shape in the discriminant canonical functions.

Table 3 represents the correlation matrix among climatic and lunar phase events on external egg quality and ranged from -0.537 to 0.574.

Table 3. Correlation matrix between climatic and lunar phase events included in the present study.

Variables	Minimum temperature	Maximum pressure	Maximum gust speed	Wind direction	Average wind speed	Sunshine hours	Rainfall	% Moon illuminated
Minimum temperature	1	-0.391	0.422	0.016	0.294	0.547	-0.047	-0.206
Maximum pressure	-0.391	1	-0.537	0.001	-0.188	0.029	-0.377	0.313
Maximum gust speed	0.422	-0.537	1	-0.215	0.303	0.181	0.574	-0.080
Wind direction	0.016	0.001	-0.215	1	0.081	0.150	-0.257	0.197
Average wind speed	0.294	-0.188	0.303	0.081	1	0.153	0.033	0.122

Sunshine hours	0.547	0.029	0.181	0.150	0.153	1	-0.455	-0.001
Rainfall	-0.047	-0.377	0.574	-0.257	0.033	-0.455	1	-0.221
% Moon illuminated	-0.206	0.313	-0.080	0.197	0.122	-0.001	-0.221	1

Results from the test of equality of group means are represented in Table 4. Variables were ranked attending to their discrimination ability, which increases with rising F values and decreasing Wilk's Lambda. Hence, variables not showing discriminating ability ($p > 0.05$) were rainfall, wind direction, maximum pressure, and the remaining moon phases (full moon, new moon, waxing crescent, waxing gibbous, and waning crescent). Moreover, the mean values of weather and moon phase variables for each egg quality-related group are shown in S1 Table.

Table 4. Results for the tests of equality of group means to test for difference in the means across egg categories.

Test of Functions	Rank	Lambda	F	GL1	GL2	p-value
Sunshine hours	1	0.691	13.950	6	187	<0.0001
Minimum temperature	2	0.874	4.476	6	187	0.000
Maximum gust speed	3	0.944	1.841	6	187	0.043

Fig 3 represents the territorial map of observations once the values of environmental factors were replaced in the two first discriminating functions to obtain x and y-axis coordinates, for the first and second dimensions (F1 and F2), respectively. Subsequently, each observation was sorted and assigned across the different egg categories.

Fig 3. Territorial map depicting the eggs considered in the canonical discriminant analysis sorted across egg weight and shape index categories. Circles represent the centroids or canonical group means.

The Mahalanobis distances between categories were computed from the centroids generated by the discriminant routine. It is based on the fact that Mahalanobis distances can be estimated through the relative distance of a given observation to the centroid of its nearest group. Fig 4 represents the Mahalanobis distances transformed into squared Euclidean distances.

Fig 4. Tree diagram based on Mahalanobis distances between environmental factors influencing egg weight and shape index.

Discriminant function cross-validation

Fig 5 reports the results obtained in the leave-one-out classification matrix. The model presented a 74.23% of correctly assignments of observations within their categories. Eggs belonging to the Rounded-3, Standard-1, Sharp-2, Sharp-1 and Standard-2 categories presented a high accuracy of correct matching (100.00, 93.55, 84.00, 79.41 and 74.36%, respectively). However, observations from the Standard–3 and Sharp–3 groups showed a moderately successful clustering rate (52.94 and 47.22%, respectively). A Press' Q value of 569.24 (N= 194; n= 144; K= 7) was obtained. Therefore, predictions were considered to be significantly better than those that would be obtained by chance at 95%.

Fig 5. Graphical representation of the proportion of correct assignments obtained using the classification and leave-one-out cross-validation matrices.

Discussion

The present work constitutes a pioneer study on several aspects. The effect of the environment on external egg quality in turkeys has been studied for the first time in this

research. Moreover, this could be the first study of the species from a layer perspective in free-range systems, as well as one of the first studies addressing a local breed. The reason for this could be a consequence of the lack of resources allocated to the research of unconventional systems and indigenous genotypes, as well as the perception of turkey as a minor layer species in developed countries. Additionally, this work could provide a deeper understanding of the main environmental agents to which outdoor layers are exposed.

The preliminary test reported three variables exhibiting multicollinearity problems. Minimum atmospheric pressure was the first variable showing redundancy. In this respect, contradictory results are found in the bibliography. All pressure measures presented multicollinearity problems in a previous study [48], while maximum pressure (instead of minimum) was found to be redundant in another study [51]. Nevertheless, multicollinearity problems found in the present study could be a consequence of including other parameters to which pressure is highly correlated, such as wind speed [52], sunlight hours, and temperature [51]. The moon phase was the second variable exhibiting redundancy. In this case, due to the lack of previous reports, the inclusion of the moon's illuminated surface could be suggested as the source of multicollinearity. Finally, the maximum temperature was the last redundant variable. Regarding this aspect, no clear conclusions can be drawn from the literature. In the study developed by Iglesias Pastrana et al. [48], all the temperature parameters included exhibited redundancy while Marín Navas et al. [51] did not find multicollinearity among temperature variables. However, these authors reported that maximum temperature was highly correlated to minimum temperature, sunlight hours, and pressure, variables that are included in the present study.

Moderate positive and negative correlations among climatic and lunar variables are depicted in Table 3. The effect of maximum gust speed on egg quality is suggested to be generally associated with squall events. This variable was positively associated with rainfall (0.574), minimum temperature (0.422), and average wind speed (0.303), and negatively associated with maximum pressure (-0.537). The same trend seems to be described for rainfall, negatively associated with sunshine hours (-0.455), maximum pressure (-0.377), and wind direction (-0.257). Contrarily, the effect of minimum temperature seems to be associated with anticyclones and showed correlation with sunshine hours (0.547), average wind speed (0.294), and maximum pressure (-0.391). In this regard, similar correlations were found in a study of climatic variables influencing diversity parameters in a horse population [51]. Lastly, the illuminated surface of the moon correlated moderately to poorly with maximum pressure (0.313), wind direction (0.197), rainfall (-0.221), and minimum temperature (-0.206). Despite not showing discriminant capacity, interesting conclusions can be drawn, such as the possible evidence of the mediation of the lunar cycle in the influence of climatic variables and also on egg quality.

The test of equality of group means reported three variables to significantly perform as explanatory variables in the analysis. Sunshine hours was the variable reporting the highest discriminating power among egg groups. Similar results were described in a study on the correlations between meteorology and fowl egg quality, in which sunshine was the most influencing parameter [53]. Photoperiod is known to directly influence external egg quality [3, 28], including egg weight, length, and width [53]. The general assumption is that, by increasing the photoperiod, higher yields are obtained, up to a certain limit [54]. Optimal light exposures to stimulate normal laying production in commercial turkey hens have been reported to be 14 to 16 hours, with negative effects occurring if 20 hours are exceeded [55]. This effect is firstly

attributed to the increased general activity that photoperiod induces in animals, including feeding activity, which translates into a greater productive performance [56]. However, several hormonal mechanisms are underlying the effect of light on poultry performance. The hypothalamus-pituitary axis of birds is particularly sensitive to light, which directly stimulates GnRH hormone production [57]. This might explain the strong effect of photoperiod on sexual maturation, rate of egg production, and timing of the ovulatory cycle in hens [57]. Photoperiod also influences birds' circadian cycle [58] and about half of the melatonin of some bird species as the Japanese quail is produced in extra-pineal glands, such as the retinae [59]. Moreover, increasing daylengths enhances the release of follicle-stimulating hormone, which causes greater follicular growth and development and stimulates ovulation [60]. Shell quality is also dependent on photoperiod, as light mediates mineral metabolism [61]. Long exposures to sunlight stimulate the synthesis of vitamin D, leading to a high accumulation of minerals in the shell [4]. This mechanism is even more accentuated in free-range systems, as direct sun exposures with natural UV radiation, alongside greater access to environment minerals, promote mineral metabolism leading to more deposition in the shell [4].

Attending to sunlight averages across egg categories, the present study reports that the heavier eggs (tercile 1) were obtained when the days were longer, while the opposite trend was observed for the lighter eggs. On the other hand, no clear effect of day length on shape index was described, but a slight positive tendency of elevated photoperiods producing standard-shaped eggs could be suggested within each weight group. In this manner, literature reports that photoperiod increases egg weight in turkey pullets during the first laying cycle [62]. However, no clear effect of day length on egg size has been described for adult turkey hens [55, 62]. This lack of results in literature could be a consequence of the comparison of different artificial light programs that exceed natural day lengths. Therefore, these results might not be comparable to

those obtained in the present study, in which animals were housed outdoors and only exposed to sunlight. This highlights the lack of studies involving turkeys in outdoor systems.

Finally, the high sensitivity to photoperiod obtained in the present study could suggest further conclusions. Indigenous genotypes are more adapted to harsh conditions and generally are more resistant to environmental fluctuations [2, 4, 31]. However, primitive genotypes preserve ancestral physiological mechanisms that were progressively eliminated through exhaustive selection, such as seasonality of production [63]. This is mainly ruled by the circadian cycle and pineal gland activity, which is severely atrophied in domestic animals compared to their wild ancestor [64]. Hence, the strong dependence on the photoperiod of this population could be evidence of the close relationship of the Andalusian turkey with the turkey's wild ancestor. For this same reason, the lack of discriminant ability of moon variables on egg quality is surprising. Since being a primitive breed with enhanced pineal activity kept outdoors, the influence of moonlight fluctuations on egg quality was expected [58, 65]. This relationship has already been observed in some wild bird species, in which the moon cycle influences the laying rate, egg formation, and shell quality [66].

Minimum temperature was the second most discriminating variable in the analysis. This is in line with the extensive literature describing the effects of temperature on poultry egg quality [28, 42, 53]. In commercial turkey, the comfort zone ranges from 12 to 28 °C [67], and temperatures above or below end thermal equilibrium, leading to a state of chronic stress [3]. Despite research studies have focused primarily on heat stress [68], cold also plays an important role in egg production and quality, with a detrimental effect on egg weight, shell quality, and internal attributes [68]. Among the reasons underlying this effect is the decline in overall activity in animals [69], including feeding activity [68]. Cold stress also decreases feed

efficiency [70, 71], as energy is lost by allocating it to increased body temperature [72]. Moreover, cold stress generates a decrease in plasma insulin and an increase in corticosterone, hormones with opposite effects on energy metabolism [68, 70, 73]. This has a negative effect on anabolic metabolism, as insulin promotes glucose mobilization and tissue utilization, which benefits production-interest traits [73]. Additionally, cool decreases birds' ability to digest minerals [71] and reduces the intestinal absorption of some nutrients [68]. Additionally, the cold-stress response causes zinc deficiency [73], which mediates calcium storage.

However, variability in external egg characteristics could also be a consequence of the effect of cold on internal attributes, such as albumen [13]. Thus, a study developed in chicken species found that thermoneutral hens laid eggs that had greater albumen depositions, which occur in the equatorial region of the egg, increasing the egg's shape index (rounded eggs) and egg weight as well [74]. Moreover, eggs from cold-stressed hens are known to have a lower albumen height and yolk weight [68], possibly due to a reduced synthesis of both yolk and albumen proteins, vitellogenin and triglycerides, as well as a water composition [71]. This is consistent with the means by egg categories of the present study, as heavier eggs were obtained at warmer minimum temperatures. On the other hand, colder minimum temperatures produced both medium and small eggs, indifferently. In the same line, shape index quality seems to improve with warmer minimum temperature, as they tend to produce standard eggs. This enhanced quality found when minimum temperatures are warmer suggests turkeys loose thermoneutrality in cold nights. This finding, together with the multicollinearity problems found in the maximum temperature variable, might prove that the Andalusian turkey population is more sensitive to cold than heat. This is evidence of the great adaptation of this breed to the extreme heat the Guadalquivir basin reaches [33, 34], and warns breeders to focus on protecting during winter nights to avoid a decline in egg quality.

Maximum gust speed was the third and last variable reporting explanatory power in the analysis. Attending to the means by classes, there is a clear tendency for egg weight to increase with gust speed. Moreover, within weight groups, as gust speed increases, the egg-shape index declines. In this connection, the effect of wind on commercial layers has widely been studied [28, 53, 75]. However, no clear conclusions are drawn in the bibliography since both positive and negative effects are reported. A study reporting the benefits of air velocity on egg production suggested that wind acted as a heat stress alleviation effect [76]. On the other hand, another study found wind speed to reduce egg weight and decrease the shape index, which is consistent with present results [53]. Wind reduces locomotor and feeding activity [75, 77], causes a negative water balance due to skin moisture losses [78], and predisposes to the onset of disease [79]. Nevertheless, in hot weather, wind acts as a relief factor and increases the laying rate [28, 75, 78]. However, studies were developed in indoor conditions, and no studies have been addressing the effect of wind on layers, even less in turkeys.

Finally, the leave-one-out cross-validation routine described an acceptable hit ratio, which seems to be more accurate as egg weight increases. Weather conditions producing light eggs categories (tercile 3) showed the worst results compared to the rest of the groups. Furthermore, the low inclusion of round eggs in the study (12 observations) and their presence in only one weight category (tercile 3), together with the high number of sharp eggs should be highlighted. This could be the consequence of the lack of a specific egg shape index standard for turkey species since the standards used worldwide for chicken were employed in the present work [34]. Thus, a possible deviation to the left of the shape indexes could be suggested in turkeys, at least in the present population, due to the consideration of a high percentage of eggs as undesirable, according to this standard.

Conclusion

Results from the present work provide pioneer information about the effect of the environment on egg quality in free-range turkey eggs and contribute to the scarce literature approaching indigenous genotypes. On the other hand, the limitations of working with external attributes call for further studies including a comprehensive analysis of internal and external traits to better understand the influence of climate and the lunar cycle on egg quality. This paper demonstrates that alternative egg production faces different environmental constraints than inland egg production and affects them in different ways. Among the explicative parameters found in the DCA, sunlight exhibited the greatest discriminant ability. This supposes an interesting finding, as outdoor systems do not implement artificial photoperiod, and egg quality is suggested to have seasonal variations. Minimum temperature was the second most explanatory variable. The fact that lower temperatures worsen egg quality, together with the problems of multicollinearity of maximum temperature, show that this population is more sensitive to cold than to heat. This evidences the great adaptation of this breed to the Mediterranean basin and its potential for future climate challenges, as well as points farmers to minimize cold exposure to save egg quality. Maximum gust speed was the last discriminating variable, exerting an overall positive effect on egg weight and shape index. Although excessive air velocity worsens egg quality in commercial rearing, the wind seemed to exert a positive effect on this population and housing, possibly acting as a heat-relieving factor. Therefore, maximum egg quality seems to be achieved during spring and summer seasons, when long days with warm nights and wind acting a relieving factor are commonly found. Both maximum gust speed and minimum temperature were explanatory variables reporting a high correlation to others, which makes them particularly suitable for the study of weather’s influence on egg

quality. Finally, acceptable hit classification was obtained in the cross-validation test, except from the lighter egg categories. Additionally, different sample sizes may suggest the need to determine a specific egg shape index standard for the turkey, as those for chicken were employed due to the lack of turkey species.

Acknowledgements

This work would not have been possible without the assistance of ACPA (Asociación de Criadores del Pavo Andaluz), IFAPA (Instituto de Investigación y Formación Agraria y Pesquera), Diputación de Córdoba, and the PAIDI AGR 218 research group.

References

1. González Ariza A, Arando Arbulu A, Navas González FJ, León Jurado JM, Delgado Bermejo JV, Camacho Vallejo ME. Data mining-based discriminant analysis as a tool for the study of egg quality in native hen breeds. Scientific Reports. 2022;12(1):15873.
2. González Ariza ANG, F.J.; Arndo Arbulu, A.; Delgado Bermejo, J.V.; Camacho Vallejo, M.E. Hen breed and variety factors as a source of variability for the chemical composition of eggs. Journal of Food Composition and Analysis. 2021;95:103673.
3. Bari M, Cohen-Barnhouse A, Campbell D. Early rearing enrichments influenced nest use and egg quality in free-range laying hens. Animal. 2020;14(6):1249-57.
4. Sözcu A, Aydın İ, Gunduz M. Comparison of Laying Performance, Egg Quality and Bone Characteristics of Commercial and Türk Laying Hen Genotypes Kept in a Free-Range System. Kafkas Universitesi Veteriner Fakultesi Dergisi. 2023;29(5).
5. Castillo A, Salvucci S, Mancini S, Serra A, Cappucci A, Schiavone A, et al. Physical and chemical characteristics of eggs from eight Italian chicken breeds. Italian Journal of Animal Science. 2024;23(1):342-7.
6. Georgiadis NM, Dimitropoulos G, Avanidou K, Bebeli P, Bergmeier E, Dervisoglou S, et al. Farming practices and biodiversity: Evidence from a Mediterranean semi-extensive system on the island of Lemnos (North Aegean, Greece). Journal of Environmental Management. 2022;303:114131.
7. González Ariza A, Arando Arbulu A, Navas González FJ, Delgado Bermejo JV, Camacho Vallejo ME. Discriminant canonical analysis as a validation tool for multivariety native breed egg commercial quality classification. Foods. 2021;10(3):632.
8. Sonaiya E. Family poultry, food security and the impact of HPAI. World's Poultry Science Journal. 2007;63(1):132-8.

9. Mottet A, Tempio G. Global poultry production: current state and future outlook and challenges. World's Poultry Science Journal. 2017;73(2):245-56.
10. Besbes B. Genotype evaluation and breeding of poultry for performance under sub-optimal village conditions. World's Poultry Science Journal. 2009;65(2):260-71.
11. Safiyu KK, Sogunle OM, Egbeyale LT, Shittu TA. An exploratory study on the effects of rearing system and plumage colour on performance, carcass characteristics and meat quality of local turkeys. International Journal of Health, Animal Science and Food Safety. 2019;6(1).
12. Kleyn F, Ciacciariello M. Future demands of the poultry industry: will we meet our commitments sustainably in developed and developing economies? World's Poultry Science Journal. 2021;77(2):267-78.
13. Portillo-Salgado R, Ruiz-Sesma B, Mendoza-Nazar P, Herrera-Haro JG, Bautista-Ortega J, Cigarroa-Vázquez FA. Analysis of quality and prediction of external and internal egg traits in Mexican native turkey hens. Emirates Journal of Food and Agriculture. 2020:647-52.
14. Crawford R. Introduction to Europe and diffusion of domesticated turkeys from the America. Archivos de zootecnia. 1992;41(154):2.
15. Thornton EK, Emery KF. The uncertain origins of Mesoamerican turkey domestication. Journal of Archaeological Method and Theory. 2017;24:328-51.
16. Portillo-Salgado R, Herrera Haro J, Bautista-Ortega J, Chay-Canul A, Cigarroa Vázquez F. Guajolote–A poultry genetic resource native to Mexico. World's Poultry Science Journal. 2022;78(2):467-82.
17. Canales A, Landi V, Martínez A, Macri M, Pizarro G, Delgado J, et al. Genetic characterization of the domestic turkey of Mexican backyard. Archivos de Zootecnia. 2019;68(264):480-7.
18. Bondoc OL, Ebron AO, Ramos AR, Santiago RC. Comparison of egg quality traits in different poultry species and breeds. Philippine Journal of Veterinary Medicine. 2020;57(2).
19. Siddiky NA. Backyard Poultry Production Systems in SAARC Member States: SAARC Agriculture Centre (SAC); 2017.
20. Uberu NP, Emmanuel-Udeozor IJ, Akuru EA, Ani AO, Okuli CE, Oyeagu CE. Heritability estimates, biometric and allometric growth traits in F1 progenies of the Nigerian local Turkeys (*Meleagris gallopova*). 2022.
21. Kokoszyński D. Guinea fowl, goose, turkey, ostrich, and emu eggs. Egg innovations and strategies for improvements: Elsevier; 2017. p. 33-43.
22. Bernini F, Bagnato A, Marelli SP, Zaniboni L, Cerolini S, Strillacci MG. Genetic diversity and identification of homozygosity-rich genomic regions in seven Italian heritage Turkey (*Meleagris gallopavo*) breeds. Genes. 2021;12(9):1342.
23. Arando A, González-Ariza A, Lupi T, Nogales S, León J, Navas-González F, et al. Comparison of non-linear models to describe the growth in the Andalusian turkey breed. Italian Journal of Animal Science. 2021;20(1):1156-67.
24. Akinwumi AO, Atandah RA, Olawuyi BS, Olagoke OC, Ojebiyi OO, Odunsi AA. Comparative Evaluation on Preference and Composition of Different Avian Egg Types. 2019.
25. Sun C, Liu J, Yang N, Xu G. Egg quality and egg albumen property of domestic chicken, duck, goose, turkey, quail, and pigeon. Poultry science. 2019;98(10):4516-21.

26. Kamal R, Dey A, Mondal K, Chandran P. Impact of environmental stressors on the performance of backyard poultry. Proceedings of the National Academy of Sciences, India Section B: Biological Sciences. 2018;88:79-84.
27. Gautron J, Dombre C, Nau F, Feidt C, Guillier L. Production factors affecting the quality of chicken table eggs and egg products in Europe. Animal. 2022;16:100425.
28. Nayak G, Behura N, Sardar K, Mishra P. Effect of climatic variables on production and reproduction traits of colored broiler breeder poultry. Veterinary World. 2015;8(4):472.
29. Mammo Mengesha MM. Climate change and the preference of rearing poultry for the demands of protein foods. 2011.
30. Amin E. Effect of crossing and seasons on egg production traits in turkeys. Egyptian Poultry Science Journal. 2014;34(1):165-85.
31. Salgado Pardo JI, Navas González FJ, González Ariza A, León Jurado JM, Galán Luque I, Delgado Bermejo JV, Camacho Vallejo ME. Study of meat and carcass quality-related traits in turkey populations through discriminant canonical analysis. Foods. 2023;12(20):3828.
32. Haunshi S, Doley S, Shakuntala I. Production performance of indigenous chicken of northeastern region and improved varieties developed for backyard farming. Indian Journal of Animal Sciences. 2009;79(9):901.
33. González Ariza A, Navas González FJ, Arando Arbulu A, León Jurado JM, Barba Capote CJ, Camacho Vallejo ME. Non-parametrical canonical analysis of quality-related characteristics of eggs of different varieties of native hens compared to laying lineage. Animals. 2019;9(4):153.
34. González Ariza A, Navas Gonzállez, F. J.;, Arando Arbulu AL, Jurado JMDB, J.V.; Camacho Vallejo, M. E. Discriminant Canonical Analysis as a tool to determine traces of endangered native hen breed introgression through egg hatchability phenomics. Animal bioscience. 2022.
35. Salgado Pardo J, Navas González FJ, González Ariza A, Arando Arbulu A, Leon Jurado J, Delgado Bermejo J, Camacho Vallejo M. Traditional sexing methods and external egg characteristics combination allow highly accurate early sex determination in an endangered native turkey breed. Frontiers in Veterinary Science. 2022;9:948502.
36. Salgado Pardo JI, González Ariza A, Navas González FJ, León Jurado JM, Díaz Ruiz E, Delgado Bermejo JV, Camacho Vallejo ME. Discriminant canonical analysis as a tool for genotype traceability testing based on turkey meat and carcass traits. Frontiers in Veterinary Science. 2024;11:1326519.
37. Anna Anandh MRJ, P.N.; Senthil Kumar, P.; Rajarajan, G.; Paramasivam, A. Effect of egg weight on egg traits and hatching performance of turkey (*Meleagris gallopavo*) eggs. Iranian Journal of Applied Animal Science. 2012;2(4):391-5.
38. Duman M, Şekeroğlu A, Yıldırım A, Eleroğlu H, Camcı Ö. Relation between egg shape index and egg quality characteristics. European Poultry Science/Archiv für Geflügelkunde. 2016;80(117).
39. Rathert TC, Uckardes F, Narinc D, Aksoy T. Comparision of principal component regression with the least square method in prediction of internal egg quality characteristics in Japanese quails. Kafkas Univ Vet Fak Derg. 2011;17:687-92.
40. Alkan S, Karabağ K, Galiç A, Karsli T, MS B. Effects of selection for body weight and egg production on egg quality traits in Japanese quails (*Coturnix coturnix japonica*) of different lines and relationships between these traits. Kafkas Üniversitesi Veteriner Fakültesi Dergisi. 2010;16(2).

41. Alkan S, Türker İ. Effects of Egg Shape Index on Egg Quality in Partridges. Ordu Üniversitesi Bilim ve Teknoloji Dergisi. 2021;11(2):140-51.
42. Nestor KE, Brown KI, Touchburn SP. Egg Quality and Poult Production in Turkeys: 1. Variation during a Seven Month Laying Period. Poultry science. 1972;51(1):104-10.
43. Mróz E, Stępińska M, Krawczyk M. Morphology and chemical composition of turkey eggs. Journal of Applied Poultry Research. 2014;23(2):196-203.
44. Jabbar A, Hameed A, Ditta YA, Riaz A. Relationship between eggs shape index and embryonic mortality. Online J Anim Feed Res. 2018;8:164-8.
45. Keranova N, Hristakieva P, Oblakova M. Mathematical approach for studying the influence of the shape index of turkey eggs and their mass on some incubation indicators. Agricultural Science. 2017;9(22):75-82.
46. Oblakova M. Phenotypic Correlation between Some Morphological Characteristics of Eggs in Basic Turkey Lines at the Age of 32 Weeks. Bulgarian Journal of Agricultural Science. 2006;12(3):483.
47. Navas González FJ, Jordana Vidal J, Pizarro Inostroza G, Arando Arbulu A, Delgado Bermejo JV. Can donkey behavior and cognition be used to trace back, explain, or forecast moon cycle and weather events? Animals. 2018;8(11):215.
48. Iglesias Pastrana C, Navas González FJ, Delgado Bermejo JV, Ciani E. Lunar Cycle, Climate, and Onset of Parturition in Domestic Dromedary Camels: Implications of Species-Specific Metabolic Economy and Social Ecology. Biology. 2023;12(4):607.
49. Zhang Q, Hu J, Bai Z. Modified Pillai's trace statistics for two high-dimensional sample covariance matrices. Journal of Statistical Planning and Inference. 2020;207:255-75.
50. Navarro JA. Multivariate statistical methods: a primer: Chapman and Hall/CRC; 2016.
51. Marín Navas C, Delgado Bermejo JV, McLean AK, León Jurado JM, Camacho Vallejo ME, Navas González FJ. Modeling Climate Change Effects on Genetic Diversity of an Endangered Horse Breed Using Canonical Correlations. Animals. 2024;14(5):659.
52. Mendelsohn R, Emanuel K, Chonabayashi S, Bakkensen L. The impact of climate change on global tropical cyclone damage. Nature climate change. 2012;2(3):205-9.
53. Dauda TO, Adetayo AO, Tiamiyu AK. Effects of weather on egg characteristics of Isa Brown layers in Ibadan, Nigeria. Animal Science Journal. 2006;77(1):117-21.
54. Lien R, Hess J, McKee S, Bilgili S, Townsend J. Effect of light intensity and photoperiod on live performance, heterophil-to-lymphocyte ratio, and processing yields of broilers. Poultry science. 2007;86(7):1287-93.
55. Siopes T. Lighting for summer egg production by turkeys: day length and light intensity. Poultry science. 2007;86(11):2413-9.
56. Davis G, Siopes T. The effect of light duration on turkey poult performance and adrenal function. Poultry Science. 1985;64(5):995-1001.
57. Lewis P, Gous R. Responses of poultry to ultraviolet radiation. World's poultry science journal. 2009;65(3):499-510.
58. Tarlow EM, Hau M, Anderson DJ, Wikelski M. Diel changes in plasma melatonin and corticosterone concentrations in tropical Nazca boobies (*Sula granti*) in relation to moon phase and age. General and comparative endocrinology. 2003;133(3):297-304.
59. Kumar V, Gwinner E, Van't Hof TJ. Circadian rhythms of melatonin in European starlings exposed to different lighting conditions: relationship with locomotor and feeding rhythms. Journal of Comparative Physiology A. 2000;186:205-15.
60. Kyere C, Annor S, Kagya-Agyemang J, Korankye O. Effect of egg size and day length on reproductive and growth performance, egg characteristics and blood profile of the Guinea fowl. Livestock Research for Rural Development. 2017;29(9).

61. Cui Y-m, Wang J, Zhang H-j, Qi G-h, Wu S-g. Effect of photoperiod on eggshell quality and quality characteristics of tibia, femur, and ulna in laying ducks. Poultry Science. 2021;100(10):101376.
62. Siopes T. Intermittent lighting increases egg weight and facilitates early photostimulation of turkey breeder hens. Poultry science. 1999;78(7):1040-3.
63. Balasse M, Tresset A. Environmental constraints on the reproductive activity of domestic sheep and cattle: what latitude for the herder. Anthropozoologica. 2007;42(2):71-88.
64. Wilkins AS, Wrangham RW, Fitch WT. The "domestication syndrome" in mammals: a unified explanation based on neural crest cell behavior and genetics. Genetics. 2014;197(3):795-808.
65. Chemineau P, Malpaux B, Brillard J-P, Fostier A. Seasonality of reproduction and production in farm fishes, birds and mammals. Animal. 2007;1(3):419-32.
66. Astheimer LB. Egg formation in Cassin's Auklet. The Auk. 1986;103(4):682-93.
67. Uemura D, Regmi P, Grimes J, Wang-Li L, Shah S. Low Airspeed Impacts on Tom Turkey Response to Moderate Heat Stress. AgriEngineering. 2023;5(4):1971-88.
68. Li D, Tong Q, Shi Z, Zheng W, Wang Y, Li B, Yan G. Effects of cold stress and ammonia concentration on productive performance and egg quality traits of laying hens. Animals. 2020;10(12):2252.
69. Akbari M, Torki M, Kaviani K. Single and combined effects of peppermint and thyme essential oils on productive performance, egg quality traits, and blood parameters of laying hens reared under cold stress condition (6.8±3 C). International journal of biometeorology. 2016;60:447-54.
70. Kucuk O, Sahin N, Sahin K, Gursu M, Gulcu F, Ozcelik M, Issi M. Egg production, egg quality, and lipid peroxidation status in laying hens maintained at a low ambient temperature (6 C) and fed a vitamin C and vitamin E-supplemented diet. Veterinární medicína. 2003;48(12):33.
71. Sahin N, Sahin K, Onderci M. Vitamin E and selenium supplementation to alleviate cold-stress-associated deterioration in egg quality and egg yolk mineral concentrations of Japanese quails. Biological Trace Element Research. 2003;96:179-89.
72. Durmuş İ, Kamanlı S. Effects of cold and heat stress on egg quality traits of a newly developed native hybrid layer. Turkish Journal of Agriculture-Food Science and Technology. 2015;3(6):44-447.
73. Sahin N, Onderci M, Sahin K. Effects of dietary chromium and zinc on egg production, egg quality, and some blood metabolites of laying hens reared under low ambient temperature. Biological trace element research. 2002;85:47-58.
74. Freitas LCdSR, Tinôco IdFF, Gates RS, Dos Santos TC, Andrade RR, Barbari M, Bambi G. Spatial Variability of External Egg Quality in Vertical Naturally Ventilated Caged Aviaries. Animals. 2023;13(4):750.
75. Sakurai H. Influence of Ambient Temperature and Wind Velocity on Egg Production of Japanese Quail. Japanese poultry science. 1983;20(2):80-6.
76. Purswell JL, Branton SL. Effect of air velocity on laying hen performance and egg quality. Transactions of the Asabe. 2015;58(3):813-7.
77. Hegelund L, Sørensen JT, Kjaer J, Kristensen IS. Use of the range area in organic egg production systems: effect of climatic factors, flock size, age and artificial cover. British poultry science. 2005;46(1):1-8.
78. Ruzal M, Shinder D, Malka I, Yahav S. Ventilation plays an important role in hens' egg production at high ambient temperature. Poultry science. 2011;90(4):856-62.

79. Elijah OA, Adedapo A. The effect of climate on poultry productivity in Ilorin Kwara State, Nigeria. International journal of poultry Science. 2006;5(11):1061-8.

Supporting information

S1 Table. Mean values of weather attributes and moon phase for each egg category.

Figure 1

Click here to access/download;Figure;Fig 1.tif

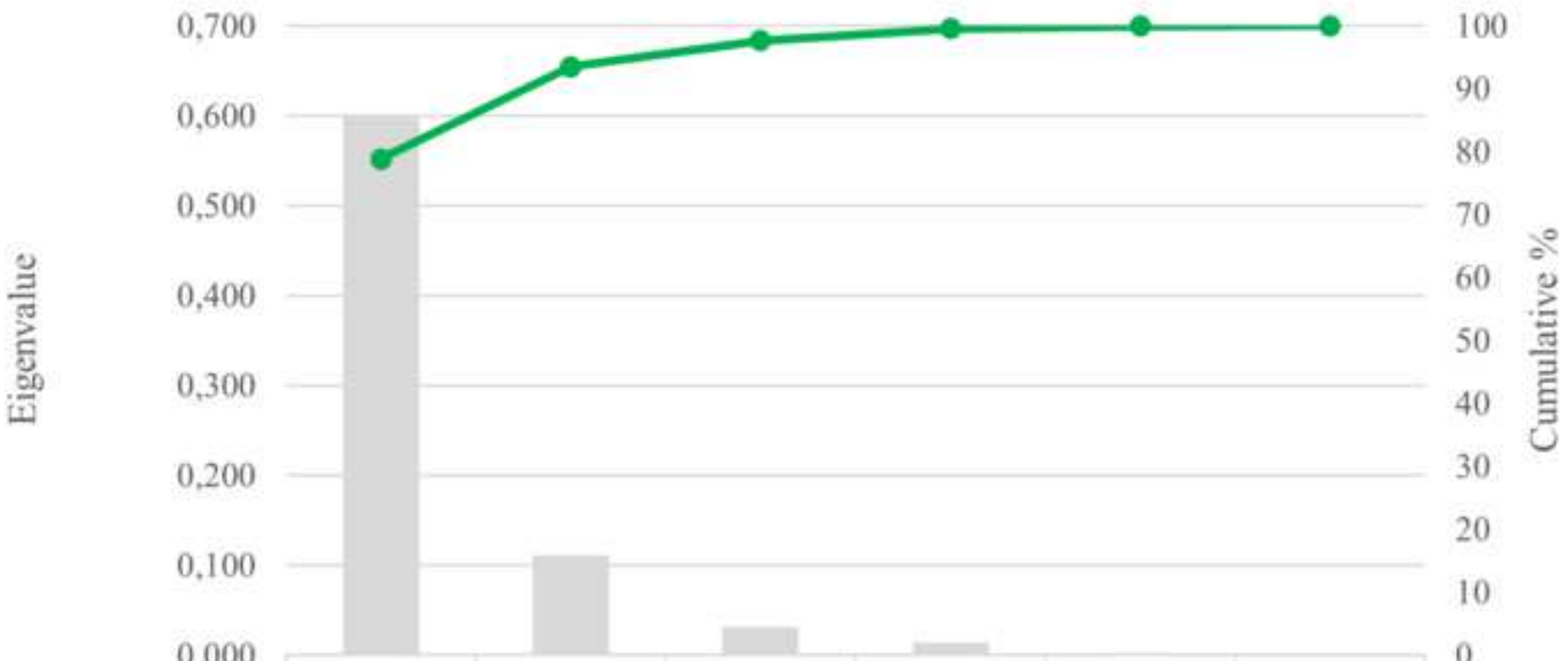

	F1	F2	F3	F4	F5	F6
Eigenvalue	0,599	0,111	0,031	0,014	0,003	0,000
Cumulative	78,84	93,52	97,66	99,55	99,95	100,00

 Click here to access/download;Figure;Fig 2.tif

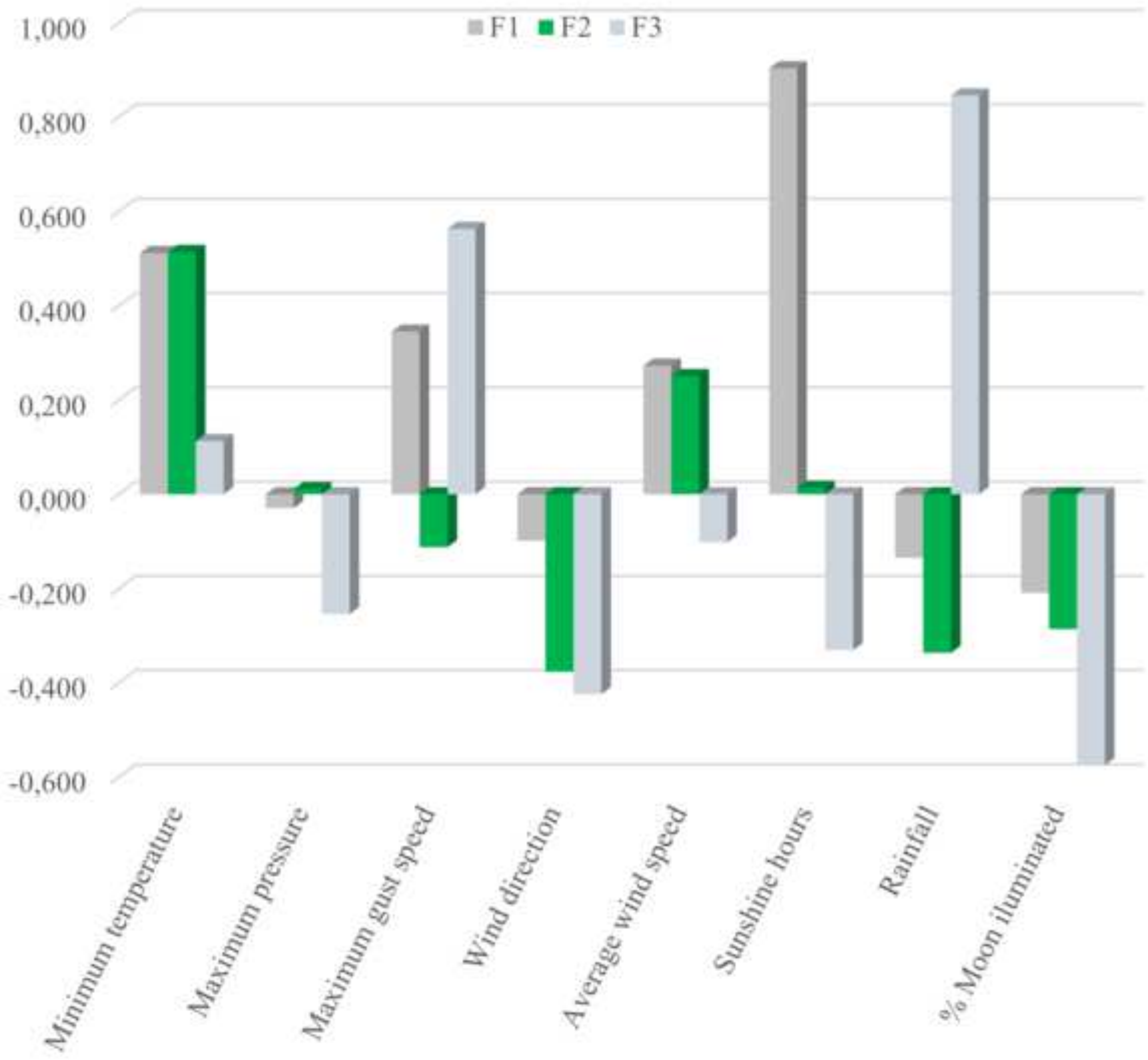

Figure 3

Click here to access/download;Figure;Fig 3.tif

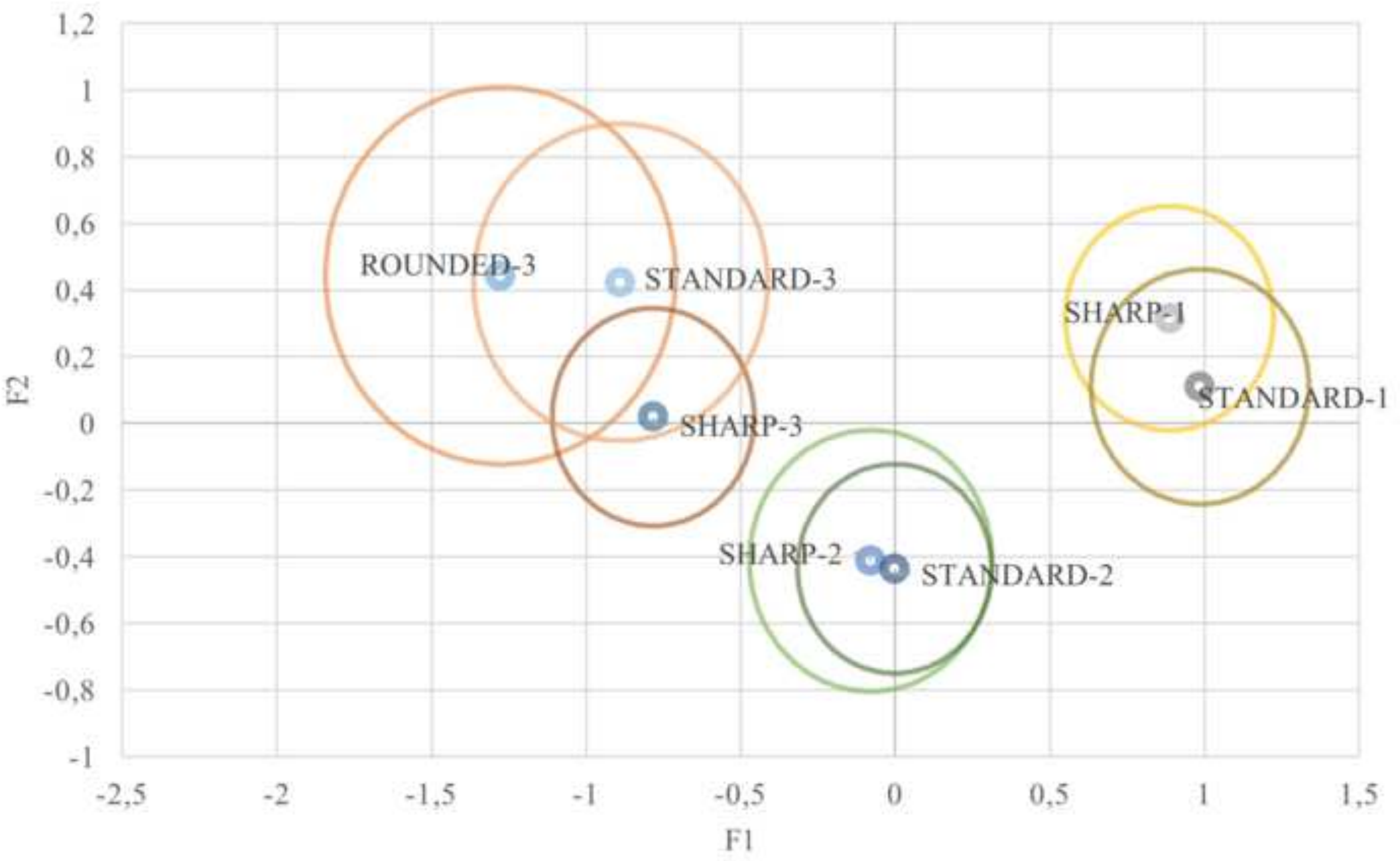

Figure 4

Click here to access/download;Figure;Fig 4.tif

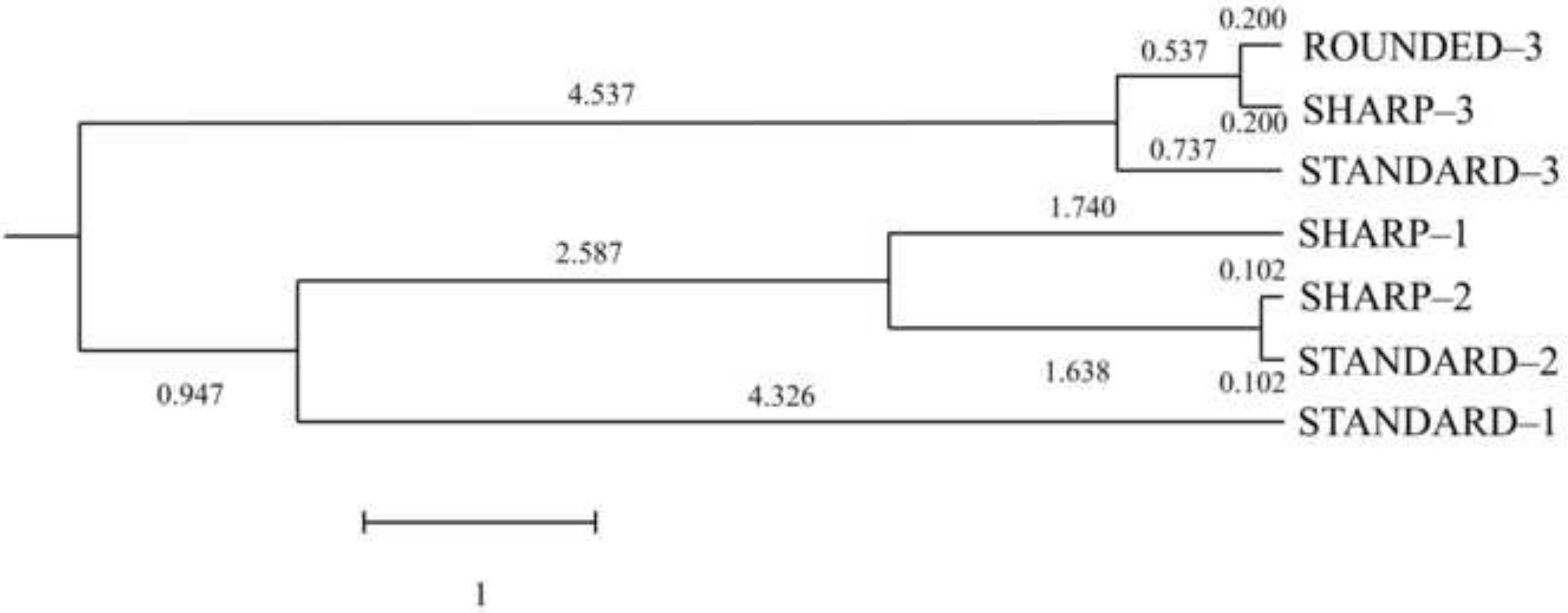

Figure 5

Click here to access/download;Figure;Fig 5.tif

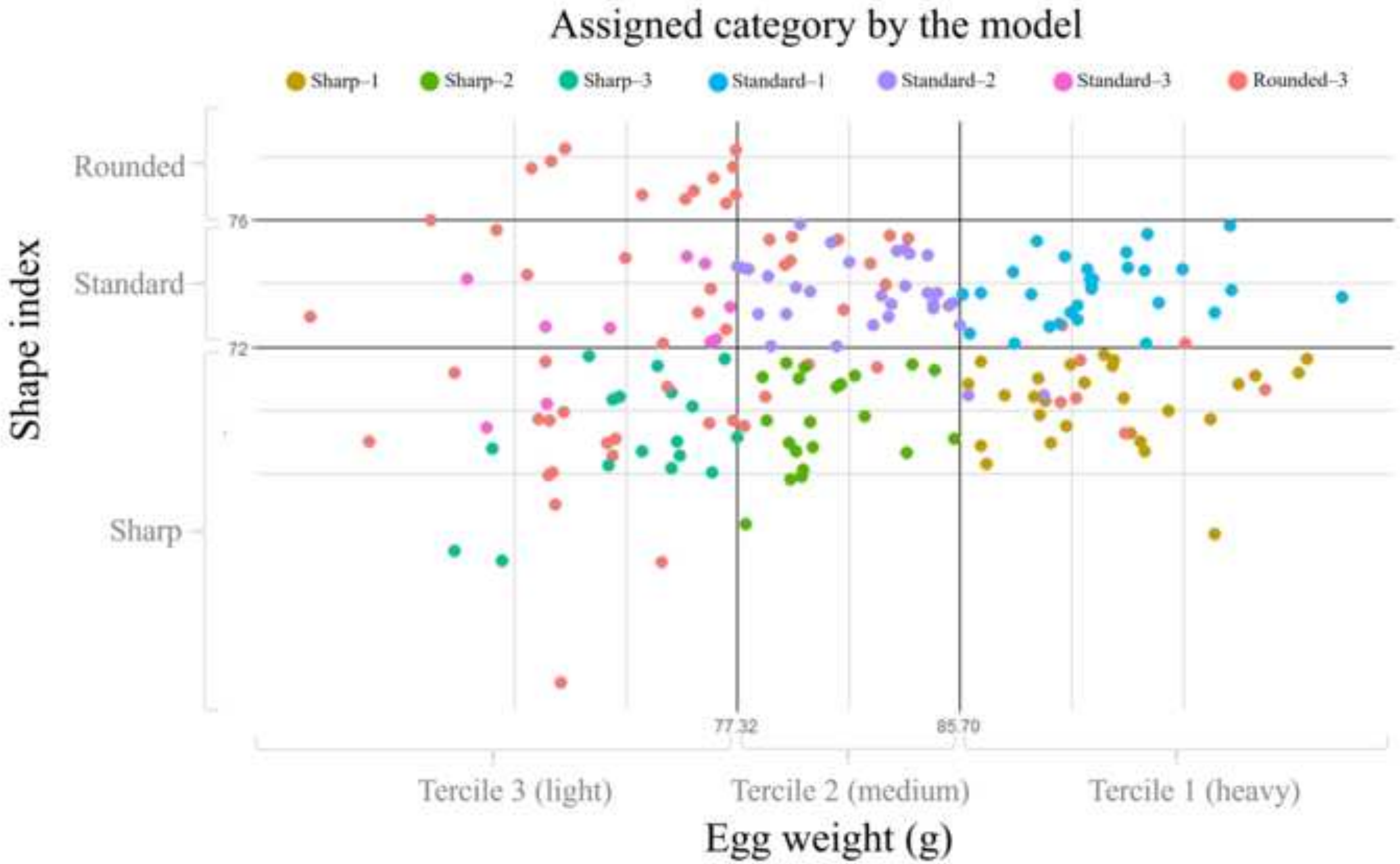

Supplementary Table S1

Click here to access/download
Supporting Information
S1_Table.docx
